# Sickness absence and disability pension among injured working-aged pedestrians - a population-based Swedish register study

**DOI:** 10.1186/s12889-021-12312-4

**Published:** 2021-12-14

**Authors:** Linnea Kjeldgård, Helena Stigson, Maria Klingegård, Kristina Alexanderson, Emilie Friberg

**Affiliations:** 1 Division of Insurance Medicine, Department of Clinical Neuroscience, Karolinska Institutet, SE-171 77 Stockholm, Sweden; 2 Division of Vehicle Safety, Mechanics and Maritime Sciences, Chalmers University of Technology, Gothenburg, Sweden; 3 Folksam Research, Folksam Insurance Group, Stockholm, Sweden

**Keywords:** Sick leave, Disability pension, Pedestrians, Fall accidents, Traffic injury, Population-based, Cross-sectional, Insurance medicine, Real-world data

## Abstract

**Background:**

The knowledge is scarce about sickness absence (SA) and disability pension (DP) among pedestrians injured in a traffic-related accident, including falls. Thus, the aim was to explore the frequencies of types of accidents and injuries and their association with SA and DP among working-aged individuals.

**Methods:**

A nationwide register-based study, including all individuals aged 16-64 and living in Sweden, who in 2010 had in- or specialized outpatient healthcare after a new traffic-related accident as a pedestrian. Information on age, sex, sociodemographics, SA, DP, type of accident, injury type, and injured body region was used. Frequencies of pedestrians with no SA or DP, with ongoing SA or full-time DP already at the time of the accident, and with a new SA spell >14 days in connection to the accident were analyzed. Crude and adjusted odds ratios (ORs) with 95% confidence intervals (CIs) for new SA were estimated by logistic regression.

**Results:**

In total, 5576 pedestrians received healthcare due to a traffic-related accident (of which 75% were falls, with half of the falls related to snow and ice). At the time of the accident, 7.5% were already on SA and 10.8% on full-time DP, while 20% started a new SA spell. The most common types of injuries were fractures (45%) and external injuries (30%). The body region most frequently injured was the lower leg, ankle, foot, and other (in total 26%). Older individuals had a higher OR for new SA compared with younger (OR 1.91; 95% CI 1.44-2.53, for ages: 45-54 vs. 25-34). The injury type with the highest OR for new SA, compared with the reference group external injuries, was fractures (9.58; 7.39-12.43). The injured body region with the highest OR for new SA, compared with the reference group head, face, and neck, was lower leg, ankle, foot, and other (4.52; 2.78-7.36).

**Conclusions:**

In this explorative nationwide study of the working-aged pedestrians injured in traffic-related accidents including falls, one fifth started a new SA spell >14 days. Fractures, internal injuries, collisions with motor vehicle, and falls related to snow and ice had the strongest associations with new SA.

## Background

Active transportation such as walking provides an opportunity for individuals to incorporate physical activity into daily life. Therefore, active transport is encouraged by different stakeholders [1–3] and in line with the UN’s global goals on sustainability [4, 5]. However, active transport is not without risks. Globally, pedestrians represent about a fifth off all fatalities within the road transport system [5, 6]. The European Commission estimates that for every person killed in road traffic crashes, an additional five suffer serious injuries [7]. In the EU, pedestrians represent the road user group with the highest proportion of accidents resulting in hospital admission [8].

Little is known about the consequences of these injuries among working-aged pedestrians in terms of sickness absence (SA) and disability pension (DP). Long-term SA or DP among injured individuals are consequences of road traffic accidents that impact the individual as well as their family, colleagues, employer, insurer, and society [9, 10]. These consequences are a major economic and political factor to consider, e.g., when establishing new policies [11].

Nevertheless, the current definition of traffic injuries [12], only includes pedestrians struck by vehicles. Several studies have indicated that if falls outdoors in public spaces were also included, the numbers of injured would be up to 35 times higher [2]. Thus, in order to avoid underestimation of injuries among pedestrians, accidents from falls sustained in the road transport system should also be included.

To get a broader understanding of SA and DP among pedestrians injured in traffic-related accidents, including falls within the road transport system, the aim of this study was to explore SA and DP among all individuals of working ages who were injured as a pedestrian, both in general and by sociodemographics, type of accident, type of injury, and injured body region.

## Methods

A population-based register study was conducted. The study population included all pedestrians aged 16-64 years, living in Sweden 31 December 2009, who in 2010 received in- or specialized outpatient healthcare due to an injury sustained in a new traffic-related accident, including falls.

Data from five nationwide registers from the following three authorities were used and linked at the individual level, using the unique personal identity number assigned to all residents in Sweden [13]:

- From *Statistics Sweden*, the Longitudinal Integration Database for Health Insurance and Labour Market Studies (LISA) was used to identify the source population of all 16-64 years old individuals living in Sweden 31 December 2009 (N=5 973 769) and information on sociodemographics (sex, age, educational level, country of birth, type of living area, and marital status).
- From the *National Board of Health and Welfare,* the in- and specialized outpatient registers were used to identify the study population as well as for medical information related to the injury. The Cause of Death Register was used to identify those who had died within the first 30 days after the accident.
- From the *Swedish Social Insurance Agency*, the register, Micro-data for Analyses of the Social Insurance (MiDAS) was used for information on dates and extent of SA and DP.

In the in- and specialized outpatient healthcare registers, the diagnoses (one main and all secondary diagnoses) and external causes of morbidity are both recorded according to the International Statistical Classification of Diseases and Related Health Problems; ICD-10 [14]. Individuals who received in- or specialized outpatient healthcare in 2010 due to an injury caused by a falls, a collision with another person, or a collision with a transport vehicle were identified by the ICD-10 codes for external causes of morbidity: “Pedestrian injured in transport accident” (V01-V09), “fall accidents, street and highway” (W00.4, W01.4, W02.4, W03.4, W04.4, W05.4, W10.4, W15.4, W17.4, W18.4, W19.4), and “striking against or bumped into by another person, street and highway” (W51.4).

These individuals (*n*=6299) will hereafter be referred to as the ‘pedestrians’ and the injuries as ‘traffic-related injuries’. The date of the accident, denoted as T_0,_ refers the date of the in- or specialized outpatient healthcare visit/hospitalization as the actual date of their accident/fall is not included in the registers.

To include only new accidents, the pedestrians who had received in- or specialized outpatient healthcare for another traffic-related accident during the three years prior to their date of the accident (T_0_) were excluded (*n*=498), leaving 5801 pedestrians. Furthermore, those 225 who did not have an injury diagnosis as a main or secondary diagnosis (ICD10: S00-T88 “Injury, poisoning and certain other consequences of external causes”) were excluded, leaving a study population of 5576 pedestrians. Those who died in immediate connection to the crash, i.e., who did not receive in- or specialized outpatient healthcare, were not included. Whereas the six pedestrians who died within 30 days of the accident were included.

Based on type of accident, the pedestrians were categorized into the following six groups: collision with pedestrian/bicyclist (V01, W03.4, W04.4, W51.4); collision with motor vehicle (V02-V06, V09.0, V09.2); unspecified (V09.1, V09.3, V09.9, W19.4); fall - snow/ice, street and highway (W00.4); fall - slipping, tripping, and stumbling, street and highway (W01.4) (*reference group*); and fall - other, street and highway (W02.4, W05.4, W10.4, W15.4, W17.4, W18.4) (including: involving ice-skates, skis, roller-skates or skateboards, involving wheelchair, on and from stairs and steps, from cliff, from one level to another, and on same level). Type of healthcare was also categorized into three groups as: only specialized outpatient healthcare (*reference group*); inpatient healthcare <3 days; and inpatient healthcare ≥3 days (the median duration of the hospital stay among those hospitalized was 3 days).

According to the patient registers, some pedestrians had up to three different healthcare visits registered on T_0_. Every visit had a main diagnosis and could also have a number of additional secondary diagnoses. The majority had only one injury diagnosis. For categorization purposes, for those with several injury diagnoses, we selected one injury diagnosis per pedestrian, in the following way: The main injury diagnosis was selected over secondary injury diagnoses, the diagnoses for inpatient healthcare over outpatient healthcare, and S00-S99 over T00-T88.

A modified version of the Barell matrix [15] was used to classify the ICD-10 codes into categories of type of injury and injured body region. Similar categorizations were used in recent studies on injuries among car occupants and bicyclists [16–19]. Type of injury was categorized into the following six groups: fracture; dislocation; sprains and strains; internal (brain, spinal cord, and other internal organs); external (open wounds, contusions, and superficial injuries) (*reference group*); and “other and unspecified”.

The injured body region was categorized into ten groups: head, face, and neck (*reference group*); vertebral column and spinal cord; torso; shoulder and upper arm; forearm and elbow; wrist, hand, and other; hip, upper leg, and thigh; knee; lower leg, ankle, foot, and other; and “other and unspecified”.

The sociodemographic covariates were categorized as: sex (women; men (*reference group*)), age group (16-24; 25-34 (*reference group*); 35-44; 45-54; 55-64 years), level of education (elementary school (≤9 years including missing); high school (9-12 years); university/college (>12 years) (*reference group*)), country of birth (Sweden (*reference group*); not Sweden), type of living area (big cities (*reference group*); medium-sized cities; small cities/villages), marital status (married (*reference group*); not married). Reference groups were chosen based on size of the groups and expected proportions with new SA, with larger groups or groups expected to have lower proportions of new SA being used as the reference.

All individuals living in Sweden, ≥16 years old, and with income from work, unemployment, or parental-leave benefits can apply for SA benefits from the Social Insurance Agency if having a disease or injury that leads to reduced work capacity [20]. The first day of a SA spell is an unreimbursed qualifying day (more days for self-employed). A physician’s certificate is required after day 7. For employees, day 2-14 are reimbursed by the employer [20]. For others, e.g., unemployed, the Social Insurance Agency administrates the benefits from the second day of SA, thus information on shorter SA spells was available for these individuals. In order not to introduce a bias, only information on SA spells >14 days was used. All individuals aged 19-64 can be granted DP if disease or injury leads to long-term or permanent work incapacity. Both SA and DP can be granted for full- or part-time (100, 75, 50, 25%) of ordinary work hours. That is, someone on part-time DP can at the same time have part-time SA. For the calculation of mean and median net days of SA (for SA spells >14 days), gross SA days were summed to whole days (e.g., two days of 50% part-time SA was counted as 1 net day).

Pedestrians were categorized into four groups regarding SA/DP at T_0_ as follows: already ongoing full-time DP; already ongoing SA; new SA; and no new SA. To be defined as already having ongoing SA, the SA spell had to have started at least five days before T_0_ and still be ongoing at T_0_. When assessing SA, any SA spells >14 days, regardless of extent (full-time, part-time) were included. Considering DP, only full-time DP was categorized as already being on DP. As it is possible to be on part-time DP and receive a new SA, the group “no new SA” includes not only pedestrians without any SA or DP but also pedestrians with ongoing part-time DP but no new SA spell >14 days. Being on part-time DP at the date of the accident was instead included as a covariate in the analyses. The pedestrians might not have received in- or specialized outpatient healthcare on the actual date of the accident, e.g., they could have sought primary healthcare first, and they might not have applied for SA benefits the first day, due to e.g., holidays, thus a window of starting days for SA in relation to T_0_ was allowed. A new SA spell in relation to the accident was defined as an SA spell that had started between 4 days before and 4 days after T_0_. The choice of the timespan of ±4 days was based on distribution of start dates of SA in relation to the date of the in- or specialized outpatient healthcare visit/hospitalization (T_0_), with significantly more SA spells starting on T_0_, but also during the days immediately before or after.

### Statistical analyses

The pedestrian’s sex, age, level of education, country of birth, type of living area, marital status, part-time DP, type of accident, type and duration of healthcare, type of injury, and injured body region were shown by SA and DP status at T_0_, using descriptive statistics.

Odds ratios (ORs) and 95% confidence intervals (CIs) for new SA were estimated by logistic regression. In these analyses, pedestrians with already ongoing SA or full-time DP were excluded (*n*=1022) as those pedestrians not were at risk of a new SA spell, leaving 4554 pedestrians. First, the ORs for new SA by the sociodemographic factors were calculated, in univariate models (crude), adjusted for sex (model 1), adjested for age (model 2), then, mutually adjusted (model 3), as well as adjusted by the accident type and injury characteristics (model 4). Then, the ORs for new SA for the characteristics of the accident were estimated, first adjusted for sociodemographic factors (model 1, 2, and 3) and then for type of accident, type of injury, and injured body region (model 4 and 5), and in model 6, 7 and 8 both the sociodemographic factors and the accident and injury characteristics were taken into consideration. These analyses were also stratified by sex. Moreover, sensitivity analyses excluding pedestrians on part-time DP were performed.

The statistical analyses were performed using SPSS (version 26) and STATA (version 14).

## Results

In total, 5576 pedestrians of working ages received in- or specialized outpatient healthcare due to injury caused by a new traffic-related accident in 2010 (Table 1). Among the injured pedestrians, a higher proportion were in the older age groups and a somewhat higher proportion were women (56.2%). A majority were born in Sweden (84.2%), were not married (65.0%), and had high school or college/university education (74.1%).

**Table 1:** Characteristics of all pedestrians aged 16-64 with a road traffic injury (including falls) in 2010 regarding sociodemographics and part-time disability pension (DP), by sickness absence (SA) and DP status at accident date (T_0_)

	All		No new SA		New SA		Ongoing SA		Ongoing full-time DP	
	n	%^a^	n	%^b^	N	%^b^	n	%^b^	n	%^b^
**Total**	5576	100	3424	61.4	1130	20.3	420	7.5	602	10.8
**Sex**										
Women	3134	56.2	1800	57.4	733	23.4	257	8.2	344	11.0
Men	2442	43.8	1624	66.5	397	16.3	163	6.7	258	10.6
**Age group, years**										
16-24	1076	19.3	950	88.3	70	6.5	33	3.1	23	2.1
25-34	701	12.6	501	71.5	116	16.5	51	7.3	33	4.7
35-44	923	16.6	577	62.5	207	22.4	81	8.8	58	6.3
45-54	1232	22.1	623	50.6	325	26.4	107	8.7	177	14.4
55-64	1644	29.5	773	47.0	412	25.1	148	9.0	311	18.9
**Level of education**										
Elementary school	1444	25.9	950	65.8	170	11.8	77	5.3	247	17.1
High school	2656	47.6	1503	56.6	640	24.1	232	8.7	281	10.6
University/college	1476	26.5	971	65.8	320	21.7	111	7.5	74	5.0
**Country of birth**										
Sweden	4695	84.2	2889	61.5	964	20.5	365	7.8	477	10.2
Not Sweden	881	15.8	535	60.7	166	18.8	55	6.2	125	14.2
**Type of living area**										
Big cities	1786	32.0	1127	63.1	331	18.5	154	8.6	174	9.7
Medium-sized cities	2213	39.7	1358	61.4	457	20.7	163	7.4	235	10.6
Small cities/villages	1577	28.3	939	59.5	342	21.7	103	6.5	193	12.2
**Marital status**										
Married	1952	35.0	1115	57.1	527	27.0	150	7.7	160	8.2
Not married	3624	65.0	2309	63.7	603	16.6	270	7.5	442	12.2
**Part-time DP at T**_**0**_										
No	5334	95.7	3299	61.8	1057	19.8	376	7.0	602	11.3
Yes	242	4.3	125	51.7	73	30.2	44	18.2	0	0.0

^a^ Column percent

^b^ Row percent

Most of the pedestrians (61.4%) did not have an ongoing SA spell >14 days or full-time DP at T_0_, nor began a new SA spell at T_0_. While 20.3% had a new SA spell, 7.5% had ongoing SA and 10.8% had ongoing full-time DP. Having a new SA spell was more common among those who were older, women, or married, and among those with ongoing part-time DP. Among those with new SA, the median number of net days of the new SA spells was 50 days, and the mean was 77 days. Most of the individuals with a new SA spell had full-time SA (88.9%). Among all pedestrians, 4.3% had ongoing part-time DP.

The two most common accident types were pedestrian falls related to snow and ice (36.3%) and falls related to slipping, tripping, and stumbling (28.0%) (Table 2). All types of falls accounted for 75.4% of the accidents. Accidents including collision with pedestrian, bicycle or motor vehicle stood for 16.5% of all accidents. Most pedestrians, 4552 (81.6%) had only specialized outpatient healthcare. Among those with inpatient healthcare ≥3 days, the proportion with a new SA spell was high (45.5%) when compared with those with inpatient healthcare <3 days (29.0%), or compared with those with only specialized outpatient healthcare (16.0%).

**Table 2:** Accident and injury characteristics of all pedestrians aged 16-64 with a road traffic injury (including falls) in 2010, by sickness absence (SA) and disability pension (DP) status at accident date (T_0_)

	All		No new SA		New SA		Ongoing SA		Ongoing full-time DP	
	n	%^a^	n	%^b^	n	%^b^	n	%^b^	n	%^b^
**Total**	5576	100	3424	61.4	1130	20.3	420	7.5	602	10.8
**Type of accident**										
Collision with pedestrian/bicyclist	245	4.4	165	67.3	33	13.5	23	9.4	24	9.8
Collision with motor vehicle	676	12.1	426	63.0	123	18.2	52	7.7	75	11.1
Unspecified	452	8.1	327	72.3	50	11.1	29	6.4	46	10.2
Fall - snow/ice	2022	36.3	1104	54.6	562	27.8	160	7.9	196	9.7
Fall - slipping, tripping, and stumbling	1563	28.0	968	61.9	272	17.4	109	7.0	214	13.7
Fall - other	618	11.1	434	70.2	90	14.6	47	7.6	47	7.6
**Healthcare**										
Only specialized outpatient care	4552	81.6	3046	66.9	728	16.0	351	7.7	427	9.4
Inpatient care <3 day	386	6.9	195	50.5	112	29.0	28	7.3	51	13.2
Inpatient care ≥3 days	638	11.4	183	28.7	290	45.5	41	6.4	124	19.4
**Type of injury**										
Fracture	2505	44.9	1083	43.2	892	35.6	253	10.1	277	11.1
Dislocation	182	3.3	120	65.9	33	18.1	14	7.7	15	8.2
Sprains and strains	849	15.2	645	76.0	76	9.0	68	8.0	60	7.1
Internal	316	5.7	216	68.4	34	10.8	20	6.3	46	14.6
External	1644	29.5	1294	78.7	89	5.4	63	3.8	198	12.0
Other and unspecified	80	1.4	66	82.5	<8	7.5	<8	2.5	<8	7.5
**Body region**										
Head, face, and neck	893	16.0	671	75.1	50	5.6	35	3.9	137	15.3
Vertebral column and spinal cord	74	1.3	40	54.1	17	23.0	11	14.9	6	8.1
Torso	285	5.1	193	67.7	37	13.0	18	6.3	37	13.0
Shoulder and upper arm	535	9.6	282	52.7	118	22.1	75	14.0	60	11.2
Forearm and elbow	402	7.2	211	52.5	121	30.1	30	7.5	40	10.0
Wrist, hand, and other	1175	21.1	709	60.3	282	24.0	81	6.9	103	8.8
Hip, upper leg, and thigh	187	3.4	104	55.6	39	20.9	16	8.6	28	15.0
Knee	536	9.6	345	64.4	72	13.4	51	9.5	68	12.7
Lower leg, ankle, foot, and other	1448	26.0	834	57.6	390	26.9	102	7.0	122	8.4
Other and unspecified	41	0.7	35	85.4	<8	9.8	<8	2.4	<8	2.4

^a^ Column percent

^b^ Row percent

Fractures and external injuries were the most common injury types, accounting for 44.9 and 29.5% of all injuries, respectively. Beginning a new SA spell was most common among pedestrians with a fracture (35.6%) and least common for external injuries (5.4%).

The most commonly injured body regions were upper and lower extremities with most injuries in the subgroup lower leg, ankle, foot, and other (26.0%) followed by the wrist, hand, and other (21.1%). Head, face, and neck stood for 16.0% of all injuries. New SA was most common among pedestrians with injuries to the forearm and elbow (30.1%).

In the analysis of ORs for new SA, only those 4554 pedestrians at risk for new SA were included. The adjusted OR for a new SA among women compared with men was 1.25 (95% CI 1.06-1.49) (Table 3). The OR for new SA was higher among older pedestrians compared to younger. When stratifying by sex and after adjusting for potential confounders, results remained similar among both women and men (data not shown).

**Table 3:** Odds ratios (ORs) and 95% confidence intervals (CIs) for new sickness absence (SA) following a road traffic injury (including falls), by sociodemographics, among the 4554 injured pedestrians without already ongoing SA or full-time disability pension

	N (%SA)	Crude	Model 1	Model 2	Model 3	Model 4
All at risk of SA		OR (95% CI)	OR (95% CI)	OR (95% CI)	OR (95% CI)	OR (95% CI)
**Sex**						
Women	2533 (28.9)	1.67 (1.45-1.91)		1.38 (1.19-1.59)	1.39 (1.20-1.61)	1.25 (1.06-1.49)
Men	2021 (19.6)	ref.		ref.	ref.	ref.
**Age group, years**						
16-24	1020 (6.9)	0.32 (0.23-0.44)	0.32 (0.23-0.43)		0.34 (0.24-0.46)	0.38 (0.27-0.54)
25-34	617 (18.8)	ref.	ref.		ref.	ref.
35-44	784 (26.4)	1.55 (1.20-2.00)	1.50 (1.16-1.95)		1.43 (1.10-1.86)	1.47 (1.10-1.97)
45-54	948 (34.3)	2.25 (1.77-2.87)	2.13 (1.67-2.72)		2.01 (1.57-2.58)	1.91 (1.44-2.53)
55-64	1185 (34.8)	2.30 (1.82-2.91)	2.13 (1.68-2.70)		2.07 (1.62-2.65)	1.68 (1.27-2.22)
**Level of education**						
Elementary school	1120 (15.2)	0.54 (0.44-0.67)	0.60 (0.48-0.74)	0.85 (0.69-1.06)	0.90 (0.72-1.12)	0.92 (0.72-1.17)
High school	2143 (29.9)	1.29 (1.10-1.51)	1.39 (1.19-1.63)	1.50 (1.28-1.76)	1.54 (1.30-1.81)	1.69 (1.41-2.03)
University/college	1291 (24.8)	ref.	ref.	ref.	ref.	ref.
**Country of birth**						
Sweden	3853 (25.0)	ref.	ref.	ref.	ref.	ref.
Not Sweden	701 (23.7)	0.93 (0.77-1.12)	0.93 (0.77-1.13)	0.89 (0.73-1.08)	0.99 (0.80-1.21)	1.09 (0.87-1.37)
**Type of living area**						
Big cities	1458 (22.7)	ref.	ref.	ref.	ref.	ref.
Medium-sized cities	1815 (25.2)	1.15 (0.97-1.35)	1.14 (0.97-1.34)	1.20 (1.01-1.42)	1.14 (0.96-1.35)	1.20 (0.99-1.46)
Small cities/villages	1281 (26.7)	1.24 (1.04-1.48)	1.25 (1.05-1.48)	1.30 (1.09-1.56)	1.23 (1.02-1.49)	1.28 (1.04-1.57)
**Marital status**						
Married	1642 (32.1)	ref.	ref.	ref.	ref.	ref.
Not married	2912 (20.7)	0.55 (0.48-0.63)	0.59 (0.51-0.67)	0.92 (0.79-1.07)	0.92 (0.79-1.07)	0.90 (0.76-1.07)
**Partime disability pension**						
No	4356 (24.3)	ref.	ref.	ref.	ref.	ref.
Yes	198 (36.9)	1.82 (1.35-2.45)	1.65 (1.22-2.22)	1.18 (0.87-1.60)	1.06 (0.78-1.44)	1.03 (0.73-1.45)

Model 1: adjusted for sex

Model 2: adjusted for age

Model 3: adjusted for age, sex, level of education, country of birth, type of living area, marital status, and part-time DP

Model 4: adjusted for factors as in model 3 as well as: type of accident, type of injury, and injured body region

Regarding the accident and injury characteristics, having had inpatient healthcare ≥3 days was associated with new SA both in the crude analysis OR 6.63 (95% CI 5.42-8.11) and after adjusting for sociodemographic factors and for accident- and injury-related factors (Model 8: OR 3.65 (95% CI 2.82-4.73)) (Table 4). Since the type of healthcare was correlated to the type of injury, we in the final model, model 7, did not include healthcare to limit the risk of over adjusting. The type of accident was, in the final model (model 7) associated with new SA for a fall related to snow and ice and for a collision with a motor vehicle compared with a fall related to slipping, tripping, and stumbling. This association for a fall related to snow and ice was also seen in the sex-stratified analyses among women, but not among men (Table 5). When examining the type of injury, fractures had a 10-times higher adjusted OR for new SA compared with external injuries. In the sex-stratified analyses, both women and men had higher ORs for new SA if having a fracture, a dislocation, or an internal injury. In addition, men had higher ORs for sprains and strains.

**Table 4:** Odds ratios (ORs) and 95% confidence intervals (CIs) for new sickness absence (SA) following a road traffic injury (including falls) among pedestrians, by accident and injury characteristics, among the 4554 injured pedestrians without already ongoing SA or full-time disability pension

		Crude	Model 1	Model 2	Model 3	Model 4	Model 5	Model 6	Model 7	Model 8
All at risk of SA	n (%SA)	OR (95% CI)	OR (95% CI)	OR (95% CI)	OR (95% CI)	OR (95% CI)	OR (95% CI)	OR (95% CI)	OR (95% CI)	OR (95% CI)
**Type of accident**										
Collision with pedestrian/bicyclist	198 (16.7)	0.71 (0.48-1.06)	0.75 (0.50-1.11)	0.99 (0.65-1.49)	1.01 (0.67-1.53)		0.87 (0.56-1.34)		1.16 (0.73-1.83)	1.23 (0.77-1.96)
Collision with motor vehicle	549 (22.4)	1.03 (0.81-1.31)	1.08 (0.85-1.38)	1.45 (1.13-1.87)	1.54 (1.19-1.99)		1.46 (1.10-1.93)		2.09 (1.55-2.81)	1.74 (1.28-2.37)
Unspecified	377 (13.3)	0.54 (0.39-0.75)	0.58 (0.42-0.80)	0.69 (0.49-0.97)	0.74 (0.52-1.03)		0.75 (0.52-1.07)		1.00 (0.69-1.45)	0.98 (0.67-1.44)
Fall - snow/ice	1666 (33.7)	1.81 (1.53-2.14)	1.79 (1.51-2.12)	1.78 (1.49-2.11)	1.78 (1.49-2.11)		1.46 (1.21-1.76)		1.43 (1.18-1.74)	1.31 (1.07-1.60)
Fall - slipping, tripping, stumbling	1240 (21.9)	ref.	ref.	ref.	ref.		ref.		ref.	ref.
Fall - other	524 (17.2)	0.74 (0.57-0.96)	0.80 (0.61-1.04)	0.97 (0.74-1.28)	1.04 (0.79-1.37)		0.75 (0.56-1.00)		1.05 (0.78-1.43)	1.08 (0.79-1.46)
**Healthcare**										
Only specialized outpatient care	3774 (19.3)	ref.	ref.	ref.	ref.	ref.	ref.	ref.		ref.
Inpatient care <3 day	307 (36.5)	2.40 (1.88-3.07)	2.54 (1.98-3.26)	2.63 (2.03-3.41)	2.86 (2.20-3.73)	2.69 (2.09-3.47)	2.90 (2.14-3.92)	2.96 (2.27-3.88)		3.20 (2.34-4.39)
Inpatient care ≥3 days	473 (61.3)	6.63 (5.42-8.11)	6.80 (5.54-8.34)	6.08 (4.93-7.51)	6.76 (5.44-8.39)	6.23 (5.07-7.66)	3.44 (2.69-4.40)	6.30 (5.06-7.84)		3.65 (2.82-4.73)
**Type of injury**										
Fracture	1975 (45.2)	11.98 (9.49-15.11)	11.70 (9.27-14.76)	10.76 (8.50-13.63)	11.01 (8.67-13.97)	11.73 (9.25-14.87)	10.02 (7.80-12.88)	11.41 (8.95-14.56)	9.58 (7.39-12.43)	6.96 (5.33-9.08)
Dislocation	153 (21.6)	4.00 (2.57-6.21)	4.16 (2.67-6.48)	3.67 (2.34-5.75)	3.76 (2.39-5.93)	4.01 (2.56-6.27)	3.03 (1.90-4.82)	4.10 (2.58-6.49)	3.17 (1.96-5.13)	2.57 (1.58-4.18)
Sprains and strains	721 (10.5)	1.71 (1.24-2.36)	1.69 (1.23-2.33)	1.69 (1.22-2.34)	1.70 (1.23-2.35)	1.78 (1.28-2.46)	1.33 (0.95-1.86)	1.86 (1.34-2.59)	1.35 (0.96-1.91)	1.40 (0.99-1.98)
Internal	250 (13.6)	2.29 (1.50-3.49)	2.30 (1.51-3.50)	2.29 (1.49-3.51)	2.37 (1.54-3.64)	2.40 (1.58-3.67)	7.13 (4.03-12.6)	2.42 (1.57-3.74)	6.49 (3.63-11.58)	3.31 (1.82-6.02)
External	1383 (6.4)	ref.	ref.	ref.	ref.	ref.	ref.	ref.	ref.	ref.
Other and unspecified	72 (8.3)	1.32 (0.56-3.13)	1.29 (0.54-3.07)	1.38 (0.57-3.30)	1.47 (0.61-3.53)	1.37 (0.58-3.26)	1.68 (0.67-4.19)	1.38 (0.57-3.35)	1.58 (0.62-4.04)	1.30 (0.49-3.44)
**Injured body region**										
Head, face, and neck	721 (6.9)	ref.	ref.	ref.	ref.	ref.	ref.	ref.	ref.	ref.
Vertebral column and spinal cord	57 (29.8)	5.70 (3.02-10.77)	5.37 (2.83-10.16)	6.17 (3.19-11.95)	5.73 (2.94-11.17)	5.37 (2.82-10.22)	3.30 (1.54-7.07)	5.14 (2.62-10.08)	2.86 (1.30-6.31)	2.31 (1.01-5.27)
Torso	230 (16.1)	2.57 (1.63-4.05)	2.61 (1.66-4.12)	2.35 (1.48-3.73)	2.33 (1.46-3.70)	2.26 (1.43-3.58)	2.50 (1.43-4.40)	2.04 (1.28-3.25)	2.03 (1.14-3.61)	2.04 (1.14-3.65)
Shoulder and upper arm	400 (29.5)	5.62 (3.92-8.04)	5.66 (3.95-8.11)	4.88 (3.39-7.04)	4.85 (3.35-7.00)	4.85 (3.38-6.97)	4.70 (2.81-7.86)	4.40 (3.04-6.38)	4.01 (2.37-6.80)	4.70 (2.75-8.03)
Forearm and elbow	332 (36.4)	7.70 (5.35-11.08)	7.17 (4.98-10.34)	7.24 (4.99-10.52)	7.15 (4.90-10.43)	6.69 (4.63-9.68)	4.50 (2.69-7.54)	6.51 (4.44-9.54)	3.95 (2.32-6.73)	4.32 (2.51-7.42)
Wrist, hand, and other	991 (28.5)	5.34 (3.88-7.34)	5.02 (3.65-6.91)	4.98 (3.60-6.89)	4.74 (3.42-6.57)	4.52 (3.27-6.24)	2.89 (1.79-4.68)	4.31 (3.10-6.01)	2.56 (1.56-4.20)	3.38 (2.05-5.60)
Hip, upper leg, and thigh	143 (27.3)	5.03 (3.16-8.03)	4.91 (3.08-7.85)	4.26 (2.64-6.86)	4.21 (2.60-6.81)	4.48 (2.80-7.18)	5.49 (2.98-10.11)	3.72 (2.29-6.03)	4.29 (2.29-8.01)	2.50 (1.29-4.85)
Knee	417 (17.3)	2.80 (1.91-4.11)	2.68 (1.82-3.94)	2.95 (2.00-4.37)	2.83 (1.91-4.19)	2.50 (1.70-3.68)	4.13 (2.42-7.06)	2.62 (1.76-3.89)	3.80 (2.19-6.58)	3.63 (2.08-6.36)
Lower leg, ankle, foot, and other	1224 (31.9)	6.28 (4.60-8.57)	6.12 (4.48-8.36)	6.62 (4.82-9.09)	6.48 (4.71-8.92)	5.57 (4.07-7.62)	4.70 (2.92-7.56)	5.99 (4.34-8.25)	4.52 (2.78-7.36)	3.98 (2.42-6.54)
Other and unspecified	39 (10.3)	1.53 (0.52-4.49)	1.47 (0.50-4.32)	1.54 (0.51-4.58)	1.63 (0.54-4.89)	1.71 (0.58-5.02)	3.94 (1.18-13.17)	1.69 (0.56-5.06)	3.90 (1.18-12.89)	3.72 (1.10-12.51)

Model 1: adjusted for sex

Model 2: adjusted for age

Model 3: adjusted for sex, age, level of education, country of birth, type of living area, marital status, and part-time DP

Model 4: adjusted for type of accident

Model 5: adjusted for type of accident, type of injury, and injured body region

Model 6: adjusted for sex, age, level of education, country of birth, type of living area, marital status, part-time DP, and type of accident

Model 7: adjusted for sex, age, level of education, country of birth, type of living area, marital status, part-time DP, type of accident, type of injury, and injured body region

Model 8: adjusted for sex, age, level of education, country of birth, type of living area, marital status, part-time DP, type of accident, type of injury, injured body region, and healthcare

**Table 5:** Odds ratios (ORs) and 95% confidence intervals (CIs) for new sickness absence (SA) following a road traffic injury (including falls) among pedestrians, by accident and injury characteristics, among the 4554 injured pedestrians without already ongoing SA or full-time disability pension, stratified by sex

	Women				Men			
		Crude	Model 2	Model 7		Crude	Model 2	Model 7
All at risk of SA	n (%SA)	OR (95% CI)	OR (95% CI)	OR (95% CI)	n (%SA)	OR (95% CI)	OR (95% CI)	OR (95% CI)
**Type of accident**								
Collision with pedestrian/bicyclist	93 (17.2)	0.60 (0.34-1.05)	0.80 (0.44-1.43)	1.02 (0.52-1.99)	105 (16.2)	1.00 (0.57-1.77)	1.30 (0.72-2.34)	1.33 (0.69-2.54)
Collision with motor vehicle	258 (24.8)	0.95 (0.68-1.31)	1.50 (1.06-2.14)	2.60 (1.71-3.94)	291 (20.3)	1.32 (091-1.91)	1.61 (1.10-2.36)	1.77 (1.13-2.75)
Unspecified	164 (14.0)	0.47 (0.29-0.75)	0.64 (0.39-1.04)	0.95 (0.55-1.64)	213 (12.7)	0.75 (0.47-1.20)	0.86 (0.53-1.39)	1.10 (0.65-1.87)
Fall - snow/ice	1068 (37.7)	1.74 (1.42-2.14)	1.74 (1.41-2.16)	1.47 (1.15-1.87)	598 (26.6)	1.88 (1.39-2.53)	1.82 (1.34-2.47)	1.37 (0.98-1.93)
Fall - slipping, tripping, stumbling	739 (25.8)	ref.	ref.	ref.	501 (16.2)	ref.	ref.	ref.
Fall - other	211 (17.1)	0.59 (0.40-0.88)	0.78 (0.52-1.18)	0.76 (0.48-1.20)	313 (17.3)	1.08 (0.74-1.58)	1.32 (0.89-1.94)	1.38 (0.90-2.12)
**Healthcare**								
Only specialized outpatient care	2113 (23.3)	ref.	ref.		1661 (14.2)	ref.	ref.	
Inpatient care <3 days	149 (45.6)	2.77 (1.97-3.88)	3.54 (2.44-5.14)		158 (27.8)	2.33 (1.60-3.39)	2.25 (1.54-3.31)	
Inpatient care ≥3 days	271 (63.8)	5.82 (4.45-7.60)	5.57 (4.20-7.39)		202 (57.9)	8.31 (6.09-11.35)	7.46 (5.42-10.27)	
**Type of injury**								
Fracture	1200 (50.5)	11.20 (8.39-14.97)	9.99 (7.42-13.46)	9.61 (6.86-13.46)	775 (36.9)	12.59 (8.49-18.68)	12.17 (8.18-18.12)	11.06 (7.14-17.13)
Dislocation	66 (22.7)	3.23 (1.71-6.09)	3.19 (1.66-6.10)	2.68 (1.33-5.39)	87 (20.7)	5.62 (2.98-10.60)	4.94 (2.59-9.42)	4.70 (2.32-9.54)
Sprains and strains	392 (8.9)	1.08 (0.70-1.67)	1.08 (0.69-1.68)	0.98 (0.61-1.57)	329 (12.5)	3.07 (1.88-5.01)	3.04 (1.85-4.99)	2.10 (1.22-3.60)
Internal	127 (10.2)	1.25 (0.67-2.36)	1.50 (0.78-2.86)	2.88 (1.26-6.58)	123 (17.1)	4.43 (2.44-8.04)	3.93 (2.15-7.18)	16.95 (7.14-40.27)
External	707 (8.3)	ref.	ref.	ref.	676 (4.4)	ref.	ref.	ref.
Other and unspecified	41 (12.2)	1.53 (0.58-4.03)	1.78 (0.66-4.82)	1.92 (0.64-5.71)	31 (3.2)	0.72 (0.09-5.44)	0.70 (0.09-5.36)	0.86 (0.10-7.30)
**Body region**								
Head, face, and neck	339 (8.0)	ref.	ref.	ref.	382 (6.0)	ref.	ref.	ref.
Vertebral column and spinal cord	36 (30.6)	5.08 (2.26-11.44)	5.56 (2.38-12.99)	1.57 (0.56-4.39)	21 (28.6)	6.24 (2.21-17.60)	6.67 (2.30-19.38)	5.47 (1.54-19.47)
Torso	103 (14.6)	1.97 (1.00-3.86)	1.86 (0.93-3.69)	1.15 (0.50-2.66)	127 (17.3)	3.27 (1.75-6.10)	2.91 (1.55-5.47)	3.50 (1.52-8.05)
Shoulder and upper arm	191 (40.3)	7.81 (4.79-12.72)	6.31 (3.83-10.40)	3.35 (1.70-6.60)	209 (19.6)	3.81 (2.21-6.55)	3.52 (2.03-6.10)	4.93 (2.11-11.50)
Forearm and elbow	219 (42.0)	8.37 (5.20-13.47)	7.10 (4.36-11.56)	2.73 (1.40-5.34)	113 (25.7)	5.39 (2.97-9.79)	6.08 (3.30-11.22)	5.74 (2.37-13.94)
Wrist, hand, and other	625 (34.1)	5.97 (3.90-9.15)	4.84 (3.13-7.47)	1.60 (0.85-3.00)	366 (18.9)	3.63 (2.21-5.96)	4.20 (2.54-6.97)	4.11 (1.85-9.15)
Hip, upper leg, and thigh	78 (26.9)	4.26 (2.25-8.04)	3.52 (1.84-6.76)	2.15 (0.94-4.90)	65 (27.7)	5.98 (3.01-11.89)	5.27 (2.62-10.61)	11.02 (4.16-29.21)
Knee	242 (16.1)	2.22 (1.32-3.74)	2.10 (1.23-3.58)	1.98 (0.98-4.00)	175 (18.9)	3.63 (2.06-6.39)	4.32 (2.42-7.72)	8.81 (3.68-21.13)
Lower leg, ankle, foot, and other	678 (34.8)	6.17 (4.04-9.43)	5.94 (3.85-9.16)	2.74 (147-5.09)	546 (28.2)	6.13 (3.87-9.72)	7.05 (4.40-11.28)	8.44 (3.86-18.47)
Other and unspecified	22 (9.1)	1.16 (0.26-5.21)	1.01 (0.22-4.65)	1.44 (0.28-7.35)	17 (11.8)	2.08 (0.45-9.65)	2.51 (0.52-12.01)	17.79 (3.17-99.80)

Model 2: adjusted for age

Model 7: adjusted for sex, age, level of education, country of birth, type of living area, marital status, part-time DP, type of accident, type of injury, and injured body region

Regarding body region, all categories had higher ORs for new SA compared to the category: head, face, and neck. Highest ORs were seen in the three categories: shoulder and upper arm, hip, upper leg and thigh, and lower leg, ankle, foot, and other. Men had the highest ORs for new SA for the two body regions hip, upper leg and thigh, and other and unspecified. Whereas women had high ORs for new SA for the three body regions shoulder and upper arm, forearm and elbow, and lower leg, ankle, foot, and other.

Excluding pedestrians with part-time DP did not change these results (data not shown).

## Discussion

This nationwide register study investigating SA and DP among the 5576 pedestrians of working ages who in 2010 had in- or specialized outpatient healthcare for a new road traffic-related accident (including falls), observed that 20% had a new SA spell (>14 days) in connection to the accident while 18% already were on SA or full-time DP. Most (75%) of the accidents were due to falls and particularly falls related to snow and ice. Falls related to snow and ice had a higher OR for new SA compared to falls related to slipping, tripping and stumbling among women but not among men.

Fractures was the most frequent type of injury among individuals included in the present study and associated with high ORs for new SA compared to external injuries. In general, among pedestrians, fractures followed by external injuries (e.g., contusion, bruise, open wound) have previously been reported to be the most common types of injuries [1, 8]. Head, lower and upper extremities have been reported to be the most frequently injured body regions [1, 8]. This is in line with our findings where lower leg, ankle, foot, and other (26%) where the most frequent injured body region, followed by wrist, hand, and other (21%), and head, face, and neck (16%). In the present study, all injured body regions had higher ORs for new SA compared to injuries to head, face, and neck. This could be due to the readiness to seek healthcare being higher if your head, face, or neck is injured, while these injuries do not necessarily lead to long (>14 days) SA (e.g. concussion).

There are notably few studies on pedestrian falls [1, 21], However, several studies show that pedestrian falls make up a larger proportion than pedestrians injured in collisions with motor vehicles [21–23]. In this study we found that 75% of the injuries were due to a fall, 8% of the injuries occurred in an unspecified accident, and only 16% of the injuries in the road traffic system were related to an accident with another road user involved. However, in the current definition of traffic accident, only accidents where a vehicle is involved are included [12]. We start all of our journeys as pedestrians, regardless of the other means of transportation taken in the journey. To be able to capture the whole picture of pedestrians’ injuries, with a holistic perspective of the entire journey, all pedestrians’ accidents are needed to be taken into consideration, not only those involving a vehicle.

It has further been shown that in pedestrian falls, the elderly are over represented (Oxley, 2018). The elderly are more likely to fall and are also more prone to get a fracture when falling [24]. In our study, including adults younger than 65 years, we observed that over half of the injured pedestrian were found in the two oldest age groups (45-54 and 55-64 year of age). These groups also had a higher proportion of new SA in connection to the accident. Equally, higher age is associated with both SA and DP [25]. Even though outdoor falls are associated with an active lifestyle [26] a higher proportion of ongoing SA and full-time DP (18.3%) was found in the present study compared to two other studies using the same source population of adults of working age in Sweden, studying SA and DP among individuals injured in a car crash (9% ongoing SA/DP) [16] and bicycle crash (10% ongoing SA/DP) [17]. This could partly be explained by the slightly older population, compared to the age distribution in the other two studies focused on bicyclists and car occupants. The individuals with ongoing SA or full-time DP are not at risk of new SA and hence were excluded from the analyses. In addition, the analyses were adjusted for age.

Vulnerable road users, such as pedestrians, bicyclists, and motorcyclists sustain severe initial injuries more frequently in the youngest and oldest age groups compared to protected road users who have their highest number of severe injuries in the age-group 25-44 years old [27]. The mean and median number of net days of the new SA spells where higher in the present study compared to above mentioned bicycle crash study [17]. This could be seen in EU where pedestrians represent the road user group with the highest proportion of accidents where the injured is admitted to hospital and also the longest average of hospital stay [8]. Therefore, it should also be of interest to study the SA and DP of pedestrians in a longer perspective as have been done for bicycle crashes [19].

In a country like Sweden, winter lasts for several months and thereby falls related with snow and ice were high in the present study. Specifically, among women falls related to snow and ice had high ORs for new SA compared to fall related to slipping, tripping and stumbling. Snow and ice have been identified as a contributing factor to higher risk of falling [21, 23]. Days with rain followed by falling temperatures or freezing rain has been found to generate three-fold higher numbers of falls [28]. This implies that local authorities should take a wider responsibility regarding improved maintenance after snow falls. Furthermore, future studies should investigate more in detail how the seasonal variations influence the injury patterns. One explanation for women being at higher risk is that they to a greater extent have osteoporosis and higher propensity to fall [24].

### Strengths and limitations

The use of information from high-quality nationwide registers with total population coverage means that all diagnoses were certified by a physician rather than self-reported, and that all could be included without dropouts [29]. We cannot rule out that sometimes external causes of morbidity and/or secondary diagnoses might not have been recorded. All pedestrian injuries, including those from road traffic accidents due to falls, severe enough to require in- or specialized outpatient healthcare were included, which is often not the case. Another strength is the large number of included individuals allowing for subgroup analyses. The high-quality register-data means that results were not hampered by recall bias.

One limitation is that we did not have data from primary healthcare, however, all the more severe injuries were included. Another limitation is that only one injury diagnosis was taken into consideration; this may have led to under- or overestimation of the impact of different injuries.

This study focuses only on pedestrians of working ages and only to the immediate consequences of the injury, in terms of SA. Long-term consequences, regarding SA and DP, also need to be investigated in future studies. To further elucidate the consequences of pedestrians’ injuries, all age groups, need to be taken into consideration. In addition, to also capture the impact of minor accidents, primary healthcare could be considered. Moreover, the impact of intoxication, comorbidities, and use of medications on the associations need to be further evaluated.

In order to provide a complete picture of the occurrence of pedestrian falls within the road traffic system, using register data and the ability to include all injuries is an advantage. The knowledge from this study can be used as basis for more specific recommendations for future policies to contribute to a safer traffic environment for pedestrians which goes beyond for example personal anti-slip devices. While it is not reasonable to believe that it is possible to completely eliminate all accidents, injuries are not unpredictable. Accordingly, evidence-based policies could lead to prevention and less severe injuries when they do incur.

## Conclusions

This nationwide register-based study of pedestrians injured in a road-traffic-related accident including falls, found that 20% had a new SA spell lasting more than 14 days in connection to the accident and that 18% already were on SA or full-time DP at the time of the accident. Most of the pedestrians’ accidents were falls and in particular falls related to snow and ice. Fractures and internal injuries had the strongest associations with new SA. Women had high ORs for new SA after being involved in a collision with motor vehicle or in a fall related to snow and ice. The later could not be seen among men.

## Data Availability

The data cannot be made publicly available, according to privacy regulations. According to the General Data Protection Regulation, the Swedish law SFS 2018:218, the Swedish Data Protection Act, the Swedish Ethical Review Act, and the Public Access to Information and Secrecy Act, data can only be made available, after legal review, for researchers who meet the criteria for access to this type of sensitive and confidential data. Readers may contact professor Kristina Alexanderson (kristina.alexanderson@ki.se) regarding the data.

## Abbreviations

DP: Disability pension
SA: Sickness absence
OR: Odds ratio
CI: Confidence interval
